# A sialyltransferases-related gene signature serves as a potential predictor of prognosis and therapeutic response for bladder cancer

**DOI:** 10.1186/s40001-023-01496-7

**Published:** 2023-11-15

**Authors:** Penglong Cao, Mingying Chen, Tianya Zhang, Qin Zheng, Mulin Liu

**Affiliations:** 1 Department of Clinical Laboratory, First Affiliated Hospital of Dalian Medical University, No. 222 Zhongshan Road, Dalian, 116011 Liaoning China; 2 Department of Biochemistry and Molecular Biology, Liaoning Provincial Core Lab of Glycobiology and Glycoengineering, College of Basic Medical Science, Dalian Medical University, 9 West Section, Lvshun South Road, Dalian, 116044 Liaoning China

**Keywords:** Bladder cancer, Sialyltransferase, Prognostic signature, Immunotherapy, Biomarker

## Abstract

**Background:**

Aberrant glycosylation, catalyzed by the specific glycosyltransferase, is one of the dominant features of cancers. Among the glycosyltransferase subfamilies, sialyltransferases (SiaTs) are an essential part which has close linkages with tumor-associated events, such as tumor growth, metastasis and angiogenesis. Considering the relationship between SiaTs and cancer, the current study attempted to establish an effective prognostic model with SiaTs-related genes (SRGs) to predict patients’ outcome and therapeutic responsiveness of bladder cancer.

**Methods:**

RNA-seq data, clinical information and genomic mutation data were downloaded (TCGA-BLCA and GSE13507 datasets). The comprehensive landscape of the 20 SiaTs was analyzed, and the differentially expressed SiaTs-related genes were screened with “DESeq2” R package. ConsensusClusterPlus was applied for clustering, following with survival analysis with Kaplan–Meier curve. The overall survival related SRGs were determined with univariate Cox proportional hazards regression analysis, and the least absolute shrinkage and selection operator (LASSO) regression analysis was performed to generate a SRGs-related prognostic model. The predictive value was estimated with Kaplan–Meier plot and the receiver operating characteristic (ROC) curve, which was further validated with the constructed nomogram and decision curve.

**Results:**

In bladder cancer tissues, 17 out of the 20 SiaTs were differentially expressed with CNV changes and somatic mutations. Two SiaTs_Clusters were determined based on the expression of the 20 SiaTs, and two gene_Clusters were identified based on the expression of differentially expressed genes between SiaTs_Clusters. The SRGs-related prognostic model was generated with 7 key genes (CD109, TEAD4, FN1, TM4SF1, CDCA7L, ATOH8 and GZMA), and the accuracy for outcome prediction was validated with ROC curve and a constructed nomogram. The SRGs-related prognostic signature could separate patients into high- and low-risk group, where the high-risk group showed poorer outcome, more abundant immune infiltration, and higher expression of immune checkpoint genes. In addition, the risk score derived from the SRGs-related prognostic model could be utilized as a predictor to evaluate the responsiveness of patients to the medical therapies.

**Conclusions:**

The SRGs-related prognostic signature could potentially aid in the prediction of the survival outcome and therapy response for patients with bladder cancer, contributing to the development of personalized treatment and appropriate medical decisions.

## Introduction

Bladder cancer, characterized by a high recurrence rate, is the sixth most prevalent and one of the most lethal malignancies worldwide with increasing incidence and mortality [1]. It can be divided into two subtypes named non-muscle invasive bladder cancer (NMIBC) and muscle-invasive bladder cancer (MIBC) according to the invasion depth and level of the bladder wall. Also, based on the differentiation, bladder cancer can be classified into low grade (grade 1 and 2) and high grade (grade 3), whose main differences are reflected in risk stratification, patients’ management and therapy outcomes [2]. Each subtype exhibits distinct biological behavior, treatment sensitivity and prognosis. Patients will suffer with the highest life-long treatment cost among all cancer patients due to the periodic cystoscopy and expensive life-term recurrence surveillance, which is mainly due to the intrinsic or acquired drug resistance [3–5]. Currently, cisplatin-based chemotherapy and immunotherapy are preferred as the first- and second-line regimens which is beneficial to only a limited number of patients [6, 7]. Effective individualized treatment is critical for better prognosis. However, there is still a lack of specific measures to distinguish patients’ outcome, as well as the sensitivity to clinical therapies. As a consequence, identifying reliable tools to estimate prognosis and drug sensitivity to guide individual-based therapy is imperative for bladder cancer.

Aberrant glycosylation signature is one of the essential mechanism leading to tumor heterogeneity and has been recognized as one of the hallmarks of cancer [8–10]. It is a typical and complex post-translational modification of proteins catalyzed by various glycosyltransferases and glycosidases. Altered patterns of glycosyltransferases are believed to play crucial roles in multiple processes related to cancer [11–13]. For instance, Wang et al. found that FUT6 inhibited the proliferation, migration, invasion and EGF-induced epithelial to mesenchymal transition of head and neck squamous carcinoma through regulating EGFR/ERK/STAT signaling pathway [14]. Hu et al. reported that overexpressed GLANT2 in non-small cell lung cancer promoted cell proliferation, migration and invasion via modifying O-glycosylation of ITGA5, as well as the activation of PI3K/Akt and MAPK/ERK pathways [15]. Liu et al. found that the suppressive effects of low expressed FUT8 in osteosarcoma growth and progression was achieved by modifying core-fucosylation levels of TNF receptors and non-canonical NF-κB signaling pathway [16]. Protein sialylation is considered as a particular alteration during tumorigenesis catalyzed by specific sialyltransferases (SiaTs) [17–19]. To date, 20 SiaTs have been identified, including ST3GAL1-6, ST6GAL1-2, ST6GALNAC1-6, and ST8SIA1-6. Evidences have shown that differentially expressed SiaTs had close linkage with cancer progression [20–22]. Liu et al. found that downregulation of ST6GAL1 was negatively correlated with liver inflammation status which could serve as an indicator for prognosis assessment of hepatocellular carcinoma [23]. Wang et al. reported that highly expressed ST6GALNAC1 in ovarian cancer promoted cell proliferation, migration, invasion, and self-renewal through Akt signaling pathway [24]. Scott et al. identified an important role for ST6GAL1 and α2,6 sialylated *N*-glycans in the progression of prostate cancer, and highlighted the opportunity to inhibit abnormal sialylation for the development of new prostate cancer [25].

Till now, there is no research on the establishment of prognostic signature based on the sialyltransferases-related genes (SRGs) for cancers. Thus, in the current study, we aimed to generate a SRGs-related prognostic model for bladder cancer by using datasets from public databases to distinguish patients’ survival status and responsiveness to clinical medical therapies, hoping to provide solid basis for individualized evaluation of outcomes and treatment selection.

## Materials and methods

### Data acquisition

The study was performed with dataset downloaded from The Cancer Genome Atlas (TCGA, https://portal.gdc.cancer.gov/) with gene expression profile, copy number variation, single-nucleotide variant and clinicopathological data (age, gender, TNM stages, and prognostic data). The TCGA-BLCA cohort, containing 414 tumor tissue samples and 19 normal bladder tissue samples, was utilized as the training group, while GSE13507 dataset, based on GPL6102 platform downloaded from Gene Expression Omnibus (GEO, https://www.ncbi.nlm.nih.gov/geo/), was applied as the validation group. Because of the public availability of bladder cancer data from online databases, no ethical approval or informed consent was required from patients in this study.

### Consensus clustering analysis and gene set variation analysis

Based on the expression levels of the 20 SiaTs (ST3GAL1-6, ST6GAL1-2, ST8SIA1-6, ST6GALNAC1-6), consensus clustering analysis was applied to clarify patients into different sialyltransferases-related clusters (SiaTs_Cluster) with k-means algorithms by using R package of “ConsensusClusterPlus”. Gene set variation analysis (GSVA, c2.cp.kegg.v7.5.symbols and c5.go.bp.v7.5.symbols) was performed to investigate the biological functions between SiaTs_Clusters with R package “GSVA”.

### Correlations between SiaTs_Clusters and the clinicopathological parameters and the outcomes of bladder cancer

Relationships between SiaTs_Clusters, clinicopathological features (age, gender, grade, TNM stage) and patients’ outcomes were explored to elucidate the significance of clusters generated by consensus clustering analysis. The comparison of the overall survival probability between SiaTs_Clusters was determined using Kaplan–Meier analysis with R packages of “survival” and “survminer”.

### Association between SiaTs_Clusters and the immune infiltration levels in bladder cancer

The infiltration levels of 22 kinds of immune cells were computed with CIBERSORT algorithm, which was also analyzed with a single sample gene set enrichment analysis (ssGSEA) algorithm.

### Identification of differentially expressed genes between SiaTs_Clusters and functional annotations

The R package of “limma” was utilized to search for the differentially expressed genes (DEGs) in distinct SiaTs_Clusters with criteria of “∣log2fold change∣”≧1 and “*P*-value” < 0.05. Gene Ontology (GO) and Kyoto Encyclopedia of Genes and Genomes (KEGG) enrichment analysis was conducted based on the DEGs with the package of “clusterProfiler”.

### Identification of gene_Clusters based on the DEGs from distinct SiaTs_Clusters

Univariate Cox regression analysis for SiaTs_Clusters related DEGs was performed to identify the overall survival-associated DEGs. TCGA-BLCA patients were divided into distinct sialyltransferases gene clusters (gene_Clusters) based on the expression level of DEGs analyzed with consensus clustering analysis by using R package “ConsensusClusterPlus”. Then, Kaplan–Meier analysis was applied to compare the overall survival in different gene_Clusters.

### Construction of the SRGs-related prognostic signature

Expression of the DEGs from different SiaTs_Clusters were standardized across bladder cancer specimens and the intersect genes were obtained. The univariate Cox regression analysis was carried out, and the survival-associated genes were retained for further analysis. Principal component analysis (PCA) was conducted to generate sialyltransferases-related gene score (SRGs_score) with the following algorithm: SRGs_score (risk score) = expression of a gene [1] × corresponding coefficient [1] + expression of a gene [2] × corresponding coefficient [2] + expression of a gene [3] × corresponding coefficient [3] + expression of a gene [4] × corresponding coefficient [4] + expression of a gene [5] × corresponding coefficient [5] + expression of a gene [6] × corresponding coefficient [6] + expression of a gene [7] × corresponding coefficient [7].

### Evaluation and validation of the SRGs-related prognostic signature

After the prognostic scoring system was established, the median value of the predicted SRGs_score was set as the cut-off. Patients with bladder cancer was divided into high- and low-risk group. Then, the comparison of the overall survival probability between high- and low-risk groups was conducted using Kaplan–Meier analysis with R packages “survival” and “survminer”. The 1-, 3- and 5-years’ ROC curve analysis was performed with R package “timeROC”, and the corresponding area under the curve (AUC) was calculated.

### Associations of risk score with immune infiltration and tumor mutation burden in bladder cancer

The RNA-seq data of TCGA-BLCA was applied to evaluate the abundance of 22 types of immune cells. Correlations between immune cells infiltration and SRGs_score were analyzed with Spearman’s correlation analysis, and the expression profile of immune checkpoint genes was performed between different SRGs_score groups. We also extracted the mutation annotation format from TCGA data to identify the mutational landscape of bladder cancer patients in SRGs_score subgroups, and the tumor mutation score for each patient was calculated and the mutation annotation format was created. The tumor stem cell features were extracted and stem cell-like characteristics of the tumors were evaluated with the transcriptome and epigenetics of the samples from RNAss file downloaded from TCGA database.

### Establishment of the predictive nomogram for bladder cancer

The clinicopathological features, including age, gender, grade, and TNM stages, were acquired from TCGA. To individualize the predicted survival probability for bladder cancer patients, a nomogram was established using clinical features and SRGs_score to assess the predictive accuracy, including 1-, 3-, and 5-years overall survival probability. We further utilized calibration curve analysis to confirm the reliability of the predictive nomogram we have established.

### Drug sensitivity analysis

The semi-inhibitory concentration values (IC50) of various chemotherapeutic agents were evaluated with “pRRophetic” package. The lower-imputed drug sensitivity represented more sensitivity to the agents, whereas the higher-imputed represented low sensitivity.

### Statistical analysis

R software (version 4.1.2), as well as relevant packages, was applied to make the statistical analysis. Correlations were evaluated with Spearman’s correlation analysis. Differences between two groups were analyzed with the independent sample *t*-tests or Mann–Whitney U tests, whereas differences between three or more groups were performed with one-way ANOVA or Kruskal–Wallis tests. The survival evaluation was carried out with Kaplan–Meier analysis. *P* < 0.05 was set as a significant.

## Results

### Genetic alterations and expression profile of SiaTs in bladder cancer

**Fig. 1:**
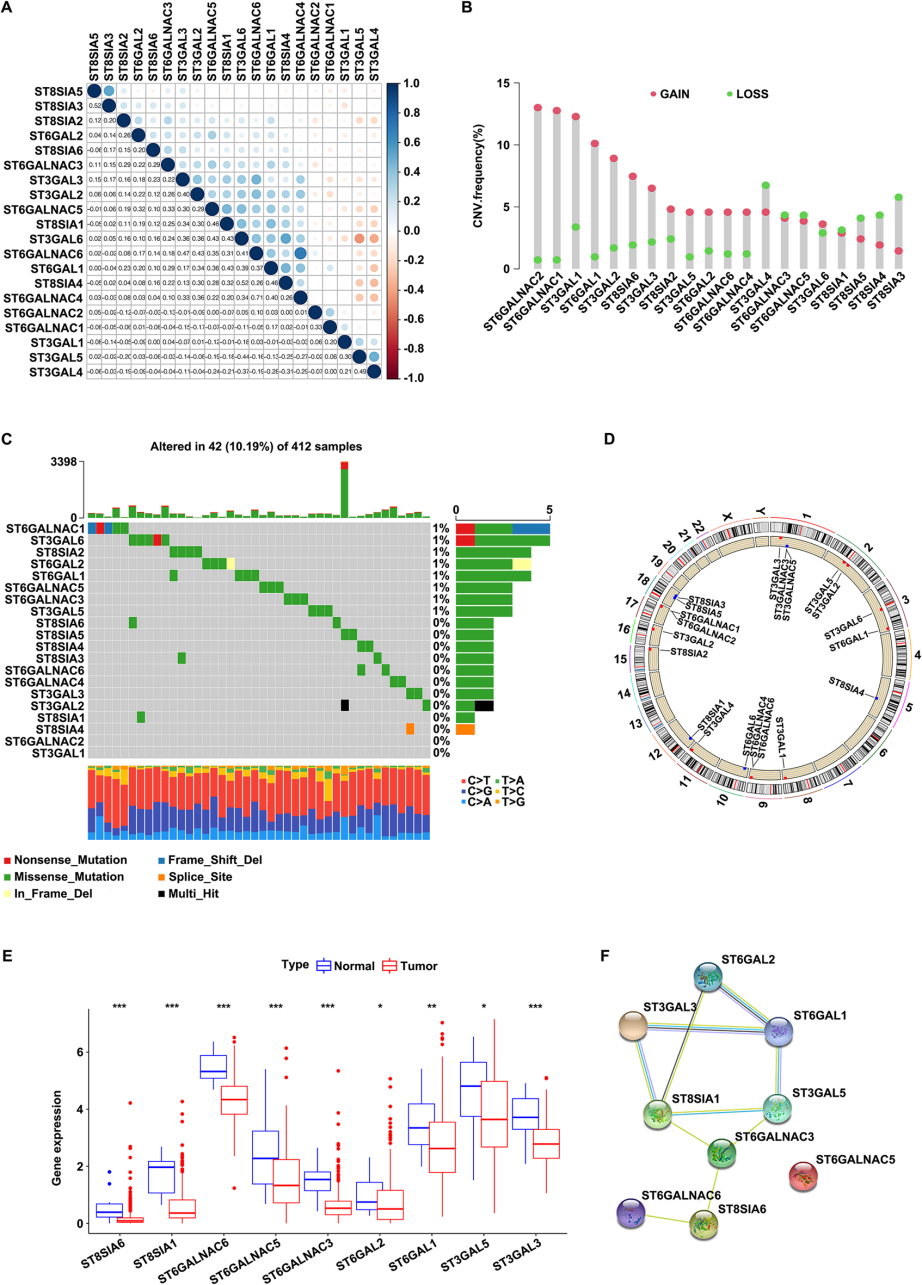
Genetic mutational landscape and the expression profile of SiaTs in bladder cancer.** A** Correlations between the 20 SiaTs by using TCGA dataset. **B** The frequency of the copy number variations of the 20 SiaTs. **C** Somatic mutations of the 20 SiaTs in 412 bladder cancer samples. **D** Locations of the CNV alterations of the 20 SiaTs on chromosomes. **E** Expression of 9 differentially expressed SiaTs in normal (blue) and tumor group (red) from TCGA-BLCA dataset. **F** Co-expression analysis of 9 differentially expressed SiaTs by using online tool-STRING. *P < 0.05, **P < 0.01, ***P < 0.001

The SiaTs family consists of 20 members (ST3GAL1-6, ST6GAL1-2, ST8SIA1-6, ST6GALNAC1-6), and abnormal expression of the SiaTs has been observed in multiple cancers. However, the relationship between the SiaTs and bladder cancer remains to be clarified. Here, the somatic mutations and copy number variations (CNV) of the SiaTs were explored with TCGA-BLCA dataset. The comprehensive landscape of the interactions between the 20 SiaTs is illustrated in Fig. 1A. As shown, the CNV alteration frequency was prevalent among the 20 SiaTs. ST6GALNACs (1–2, 4, 6), ST3GALs (1–3, 5), ST6GALs (1–2), and ST8SIAs (2, 6) represented a general frequency of CNV gain, while ST3GAL4, ST8SIAs (3–5) had a widespread frequency of CNV loss (Fig. 1B). Among the 412 bladder cancer samples, 42 (10.19%) experienced mutation in the 8 SiaTs which were ST6GALNAC1, ST3GAL6, ST8SIA2, ST6GAL2, ST6GAL1, ST6GALNAC5, ST6GALNAC3, and ST3GAL5 (Fig. 1C). Locations of the CNV alteration of the 20 SiaTs on chromosomes are shown in Fig. 1D. When compared to normal, 9 SiaTs showed decreased expression in tumor, including ST8SIAs (1, 6), ST6GALNACs (3, 5–6), ST6GALs (1–2), and ST3GALs (3, 5) (Fig. 1E), and the interactions between the 9 differentially expressed SiaTs was constructed by using STRING (Fig. 1F). The genetic heterogeneity and altered expression profile of the SiaTs suggested that SiaTs might play critical roles in the occurrence and progression of bladder cancer.

### Identification of SiaTs_Clusters and the correlations with biological functions in bladder cancer

**Fig. 2:**
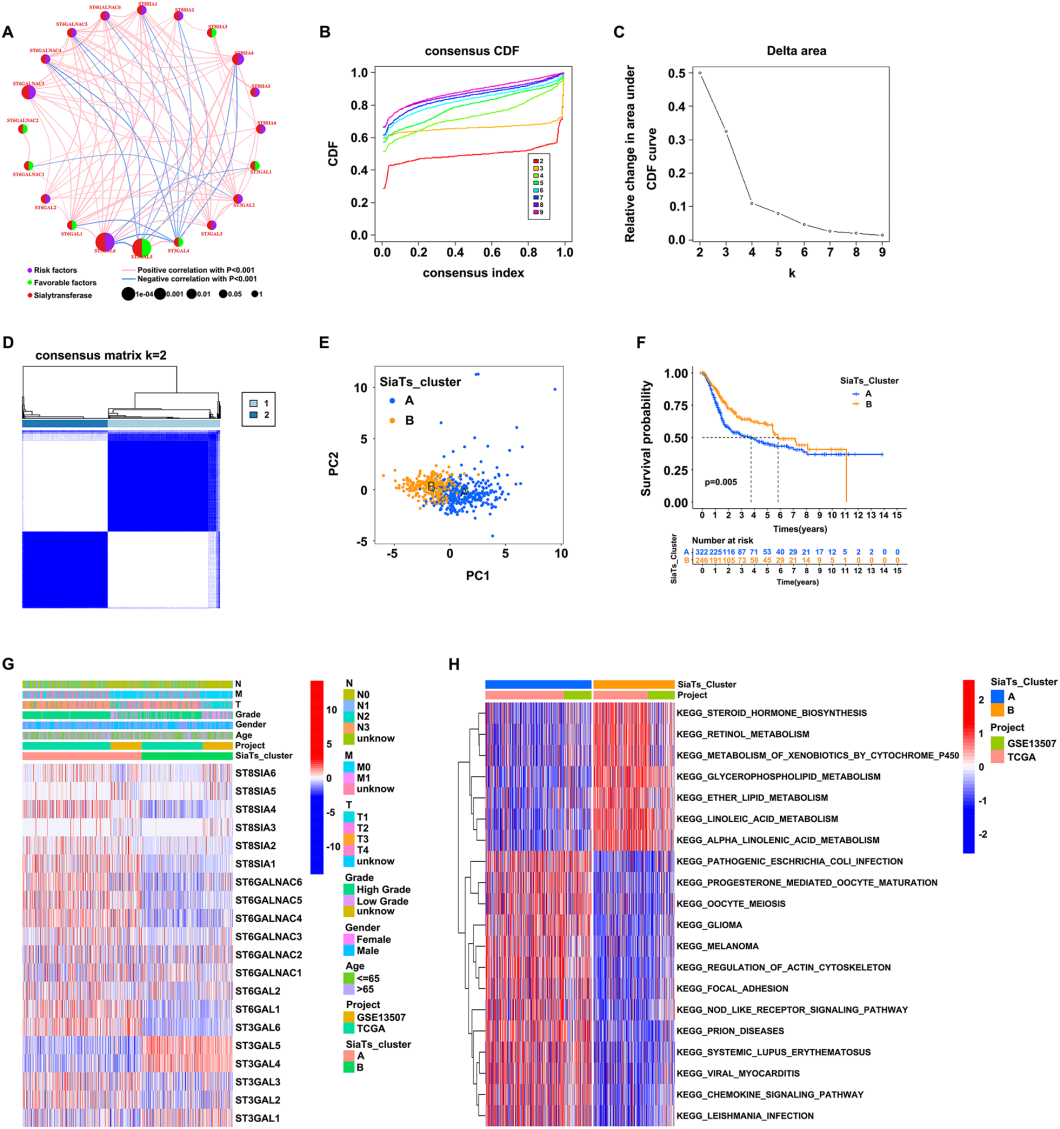
Identification of SiaTs_Clusters and the clinicopathological features of the two distinct subtypes. **A** Prognostic value and gene interactions of the 20 SiaTs for patients with bladder cancer. **B** Consensus clustering cumulative distribution function (CDF) for *k* = 2–9. **C** The area under the CDF curve for *k* = 2–9. **D** Patients were divided into two clusters (SiaTs_Cluster A and SiaTs_Cluster B) according to the consensus clustering matrix (*k* = 2). **E** Principal component analysis for the distribution of the expression of SiaTs. **F** Comparison of the survival probability of bladder cancer patients in the two SiaTs_Clusters. **G** Heatmap of the relationships between SiaTs_Clusters and the clinical characteristics, including age, gender, grade, and TNM stage. **H** GSVA enrichment analysis of the two clusters both with TCGA and GSE13507 datasets

The regulator network showed the interactions between the 20 SiaTs, regulator relation, as well as their prognostic value for bladder cancer (Fig. 2A). Patients were classified into two subgroups (SiaTs_Cluster A and SiaTs_Cluster B) analyzed with the ConsensusClusterPlus R package based on the expression level of the 20 SiaTs with the optimal clustering variable of two (*k* = 2) (Fig. 2B–D). The principal component analysis indicated that the SiaTs-based classification pattern could classify patients with bladder cancer into these two subgroups (Fig. 2E). SiaTs_Cluster A had a particularly outstanding overall survival benefit compared with SiaTs_Cluster B (*P* = 0.005) (Fig. 2F). Moreover, a heatmap was generated representing the correlations between the SiaTs_Clusters and the clinical features of bladder cancer patients (age, gender, grade, TNM stage) (Fig. 2G), as well as the GSVA enrichment analysis of the biological differences (Fig. 2H). SiaTs_Cluster A was mostly enriched in the pathogenic *Escherichia coli* infection, progesterone-mediated oocyte maturation, oocyte meiosis, glioma, melanoma, regulation of actin cytoskeleton, focal adhesion, nod like receptor signaling pathway, prion diseases, systemic lupus erythematosus, viral myocarditis, chemokine signaling pathway, and leishmania infection; whereas SiaTs_Cluster B was enriched in the steroid hormone biosynthesis, retinol metabolism, metabolism of xenobiotics by cytochrome P450, glycerophospholipid metabolism, ether lipid metabolism, linoleic acid metabolism, and alpha linoleic acid metabolism.

**Fig. 3:**
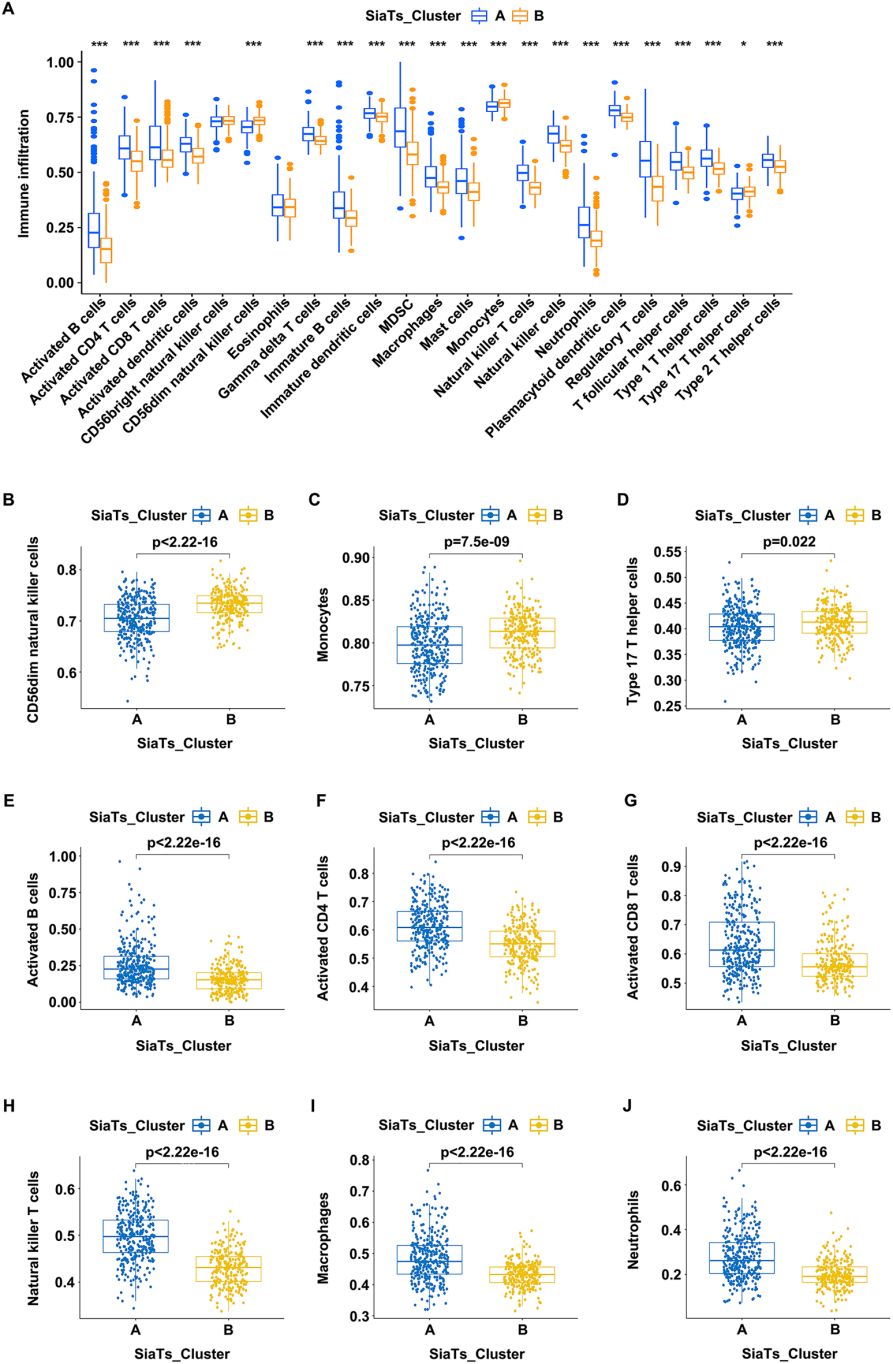
Correlations between the two subgroups and the abundance of immune infiltration in bladder cancer.** A** The abundance of immune infiltration between SiaTs_cluster A (blue) and SiaTs_cluster B (yellow). **B**–**J** Differences in the abundance of immune infiltration between the two subgroups, including CD56dim natural killer cells (**B**), monocytes (**C**), type 17 T helper cells (**D**), activated B cells (**E**), activated CD4 T cells (**F**), activated CD8 T cells (**G**), natural killer T cells (**H**), macrophages (**I**) and neutrophils (**J**). *P < 0.05, ***P < 0.001

To further analyze the differential level of the immune infiltration between the two clusters, the abundance of 22 kinds of the immune cells were included with the ssGSEA analysis. Among them, 21 types of the immune cells showed statistically difference between SiaTs_Cluster A and SiaTs_Cluster B, with significant differences in the immune cell compositions (Fig. 3A). SiaTs_Cluster A exhibited a greater enrichment of activated B cells (Fig. 3E), activated CD4 T cells (Fig. 3F), activated CD8 T cells (Fig. 3G), natural killer T cells (Fig. 3H), macrophages (Fig. 3I), and neutrophils (Fig. 3J), etc.; while CD56dim natural killer cells (Fig. 3B), monocytes (Fig. 3C) and type 17 T helper cells (Fig. 3D) were enriched in SiaTs_Cluster B. According to these findings, the SiaTs represented crucial relationship with immune infiltration, which might serve as a potential contributor to bladder cancer.

### Identification of differentially expressed genes and the construction of the SRGs-related prognostic signature

**Fig. 4:**
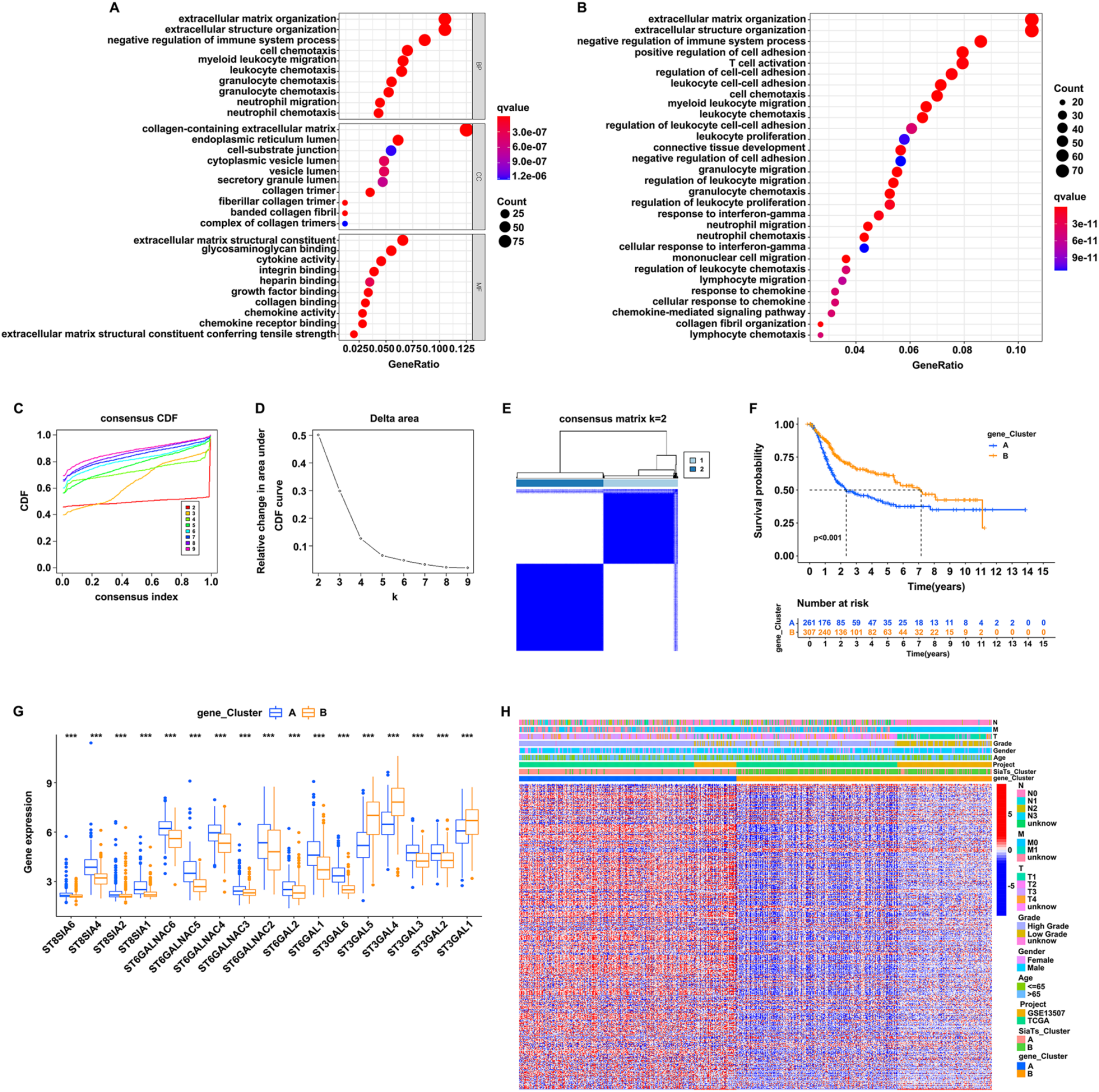
Identification of the two gene_Clusters. **A** GO enrichment analysis of the DEGs between SiaTs_Clusters. **B** KEGG pathway analysis of DEGs. **C** CDF for *k* = 2–9. **D** Relative change in area under the CDF curve from *k* = 2–9. **E** Patients were divided into two gene_Clusters according to the consensus clustering matrix (*k* = 2). **F** Comparison of the survival probability of patients with bladder cancer between gene_Cluster A (blue) and B groups (yellow). **G** Expression of the 20 SiaTs in gene_Cluster A (blue) and B groups (yellow). **H** Clinical characteristics in the two gene_Cluster groups both in TCGA and GSE13507 datasets

After the construction of SiaTs_Clusters and correlation analysis with biological functions, 372 DEGs were identified between SiaTs_Cluster A and SiaTs_Cluster B (∣log2fold change∣≧1, *P* < 0.05). The functional enrichment analysis was performed, and the top 10 enriched GO terms of biological process (BP), cellular component (CC), and molecular function (MF) were shown (Fig. 4A). It was found that the DEGs enriched in GO pathways were mainly associated with extracellular matrix organization, extracellular structure organization, collagen-containing extra cellular matrix and extracellular matrix structural constituent. In addition, KEGG enrichment analysis was also conducted, suggesting that the DEGs were mostly enriched in pathways related to extracellular matrix organization, extracellular structure organization, and negative regulation of immune system process (Fig. 4B). Then, the consensus clustering algorithm was utilized to classify patients into distinct subtypes (gene_Cluster A and gene_Cluster B) according to the expression of 372 DEGs, with the optimal clustering variable of two (*k* = 2) (Fig. 4C–E). Gene_Cluster B demonstrated better outcome than gene_Cluster A (Fig. 4F). The expression of the 20 SiaTs in these two gene_Clusters were further explored. We found that the expression of 17 kinds of the SiaTs showed significant, among which 14 were elevated (ST8SIA6, ST8SIA4. ST8SIA2, ST8SIA1, ST6GALNAC6, ST6GALNAC5, ST6GALNAC4, ST6GALNAC3, ST6GALNAC2, ST6GAL2, ST6GAL1, ST3GAL6. ST3GAL3, and ST3GAL2) in gene_Cluster A while 3 were increased (ST3GAL5, ST4GAL4, and ST3GAL1) in gene_Cluster B (Fig. 4G). The relationships between gene_Clusters and the clinical manifestations were investigated, and different characteristic genes were illustrated (Fig. 4H).

**Fig. 5:**
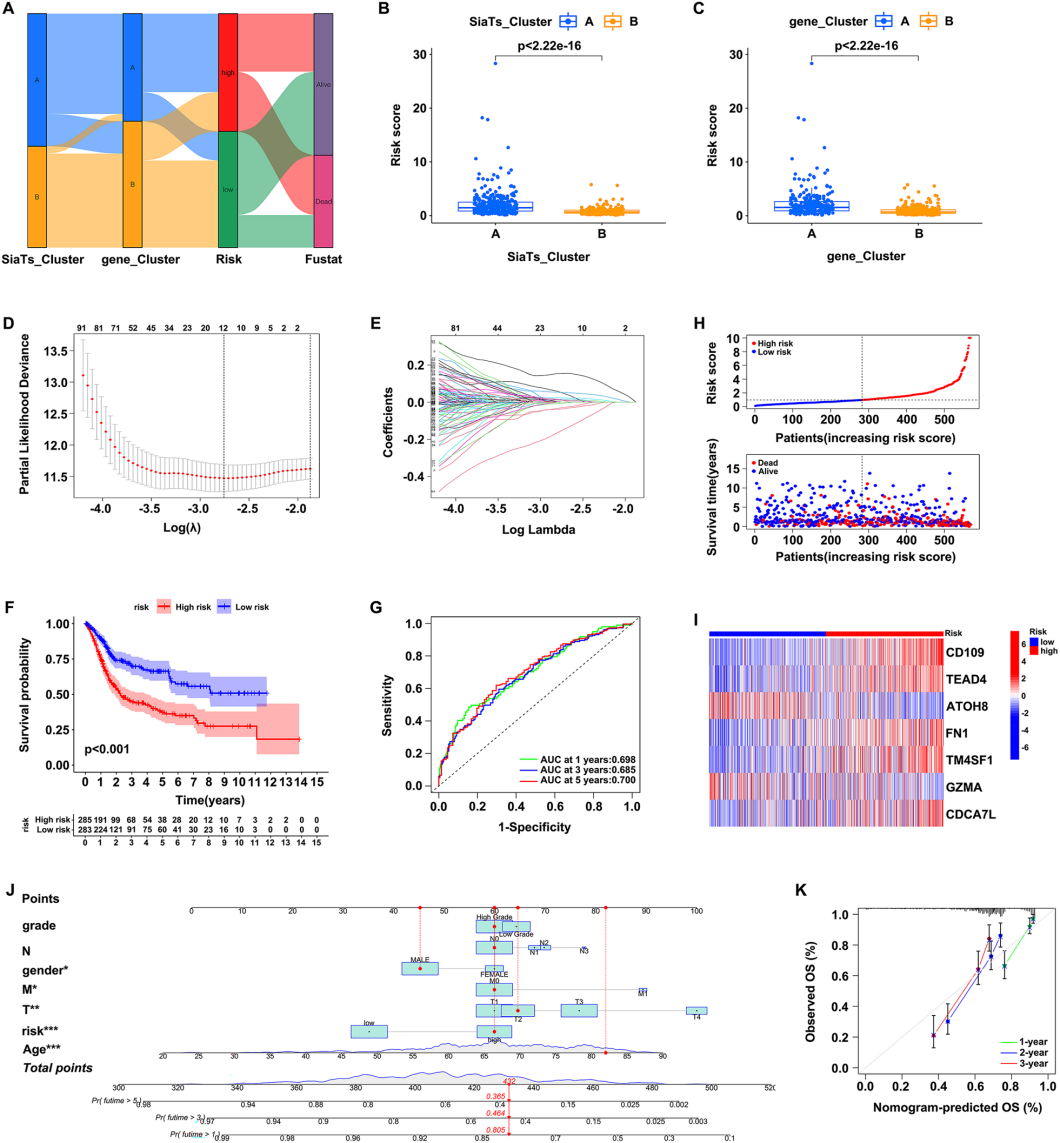
Construction of the SRGs-related prognostic signature and the validation nomogram. **A** Relationships between the SiaTs_Cluster, gene_Cluster, risk group and the survival outcomes visualized by Sankey diagram. **B** Comparison of the risk score between SiaTs_Cluster A (blue) and B groups (yellow). **C** Comparison of the risk score between gene_Cluster A (blue) and B groups (yellow). **D** LASSO analysis on the seven candidate SRGs. **E** Cross-validation in the LASSO regression. **F** Kaplan–Meier curve for overall survival between high- (red) and low-risk groups (blue). **G** Time-dependent receiver operating characteristic curve analysis of the prognostic model in predicting 1-, 3-, and 5-years survival rates of bladder cancer patients. **H** The distribution of the risk score and the patient survival outcome plots. **I** Heatmap of the 7 signature genes’ expression in the prognostic model. **J** Nomogram constructed based on the risk score and clinical characterization to predict the overall survival of bladder cancer patients. **K** Calibration plot for the internal validation of the nomogram. *P < 0.05, **P < 0.01. ***P < 0.001

The Sankey diagram was applied to visualize the correlation between the SiaTs_Cluster, gene_Cluster, risk score (SRGs_score), and the outcomes (Fig. 5A). It was found that the risk score was significantly higher in SiaTs_Cluster A (Fig. 5B) and gene_Cluster A (Fig. 5C), supporting our previous findings that SiaTs_Cluster B and gene_Cluster B had better outcomes. In order to eliminate the influence of overfitting, least absolute shrinkage and selection operator (LASSO) Cox regression analysis was performed, and the best prognostic genes were identified, which were CD109, TEAD4, FN1, TM4SF1, CDCA7L, ATOH8, and GZMA (Fig. 5D, E). The risk score was calculated based on the expression of seven genes, as well as their coefficients, and the formula was established: Risk score = (0.222 × expression of CD109) + (0.216 × expression of TEAD4) + (− 0.147 × expression of ATOH8) + (0.097 × expression of FN1) + (0.106 × expression of TM4SF1) + (− 0.364 × expression of GZMA) + (0.124 × expression of CDCA7L). Patients with bladder cancer were divided into high- and low-risk categories based on the median risk score. According to the Kaplan–Meier plot, patients with low risk tended to have a better prognosis and longer survival time (Fig. 5F). Then, time-dependent ROC curves were constructed to evaluate the predictive ability of the constructed prognostic model in predicting 1-, 3-, and 5-years survival rates for bladder cancer patients, with AUC of 0.698 after 1 year, 0.685 after 3 years, and 0.700 after 5 years (Fig. 5G), suggesting the model was a good predictor. The distribution of the risk score and the patient survival outcomes revealed that the mortality rate of patients was elevated as the risk score increased (Fig. 5H). We also evaluated the expression of the seven genes in the risk group which found that five of them (CD109, TEAD4, FN1, TM4SF1, and CDCA7L) were upregulated while two (ATOH8 and GZMA) were downregulated in the high-risk group (Fig. 5I). These results demonstrated that the SRGs-related prognostic model could effectively predict the prognosis of patients with bladder cancer. Moreover, a nomogram based on the risk score and the clinical characteristics consisting of age, gender, grade, and TNM stages for predicting the patients’ overall survival (OS) was generated. As indicated, the risk score, age, gender, T stage and M stage were significantly correlated with the outcome of bladder cancer patients (Fig. 5J), and the calibration curve for internal validation of the nomogram showed good consistency between the nomogram-predicted OS and the observed actual OS of 1, 2, and 3 years (Fig. 5K).

### Associations between the risk score and the abundance of immune infiltration and immune checkpoint genes

**Fig. 6:**
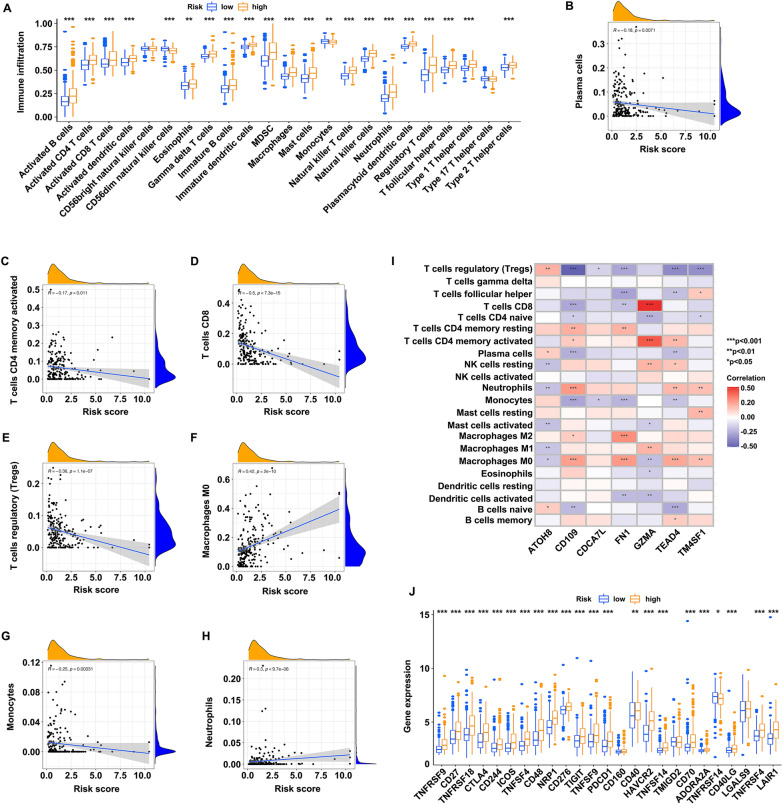
Evaluation of the abundance of the immune infiltration and immune checkpoint genes between high- and low-risk groups.** A** Differential abundance of the immune infiltration in low- (blue) and high-risk groups (yellow). **B–H** Associations between the risk score and immune cells, including plasma cells (**B**), CD4 activated memory T cells (**C**), CD8 T cells (**D**), regulatory T cells (**E**), M0 macrophages (**F**), monocytes (**G**), and neutrophils (**H**). **I** Relationships between ATOH8, CD109, CDCA7L, FN1, GZMA, TEAD4, TM4SF1 and the immune infiltration. **J** Expression of the immune checkpoint genes between low- (blue) and high-risk groups (yellow)

ssGSEA algorithm was utilized to estimate the infiltration levels of immune cells. Differential abundance of immune infiltration in high- and low-risk groups is shown in Fig. 6A. Results indicated that the high-risk group had more immune cells present. The plasma cells (Fig. 6B), T cells CD4 memory activated (Fig. 6C), T cells CD8 (Fig. 6D), T cells regulatory (Tregs) (Fig. 6E) and monocytes (Fig. 6G) were negatively correlated with the risk score. On the contrary, the macrophages M0 (Fig. 6F) and neutrophils (Fig. 6H) were positively correlated with the risk score. The relationships between the seven genes and the infiltration levels of 22 types of immune cells were detected (Fig. 6I), as well as the correlations between the risk score and the expressions of 25 kinds of immune checkpoint genes (ICIs) (Fig. 6J). A total of 22 ICIs were differentially expressed, among which the majority of them were upregulated in the high-risk group (TNFRSF9, CD27, TNFRSF18, CTLA4, CD244, ICOS, TNFSF4, CD48, NRP1, CD276, TIGIT, TNFSF9, PDCD1, CD40, HAVCR2, TNFSF14, CD70, ADORA2A, CD40LG, TNFRSF4, and LAIR1), whereas only TNFRSF14 was downregulated.

### Characteristics of the SRGs-related prognostic signature

**Fig. 7:**
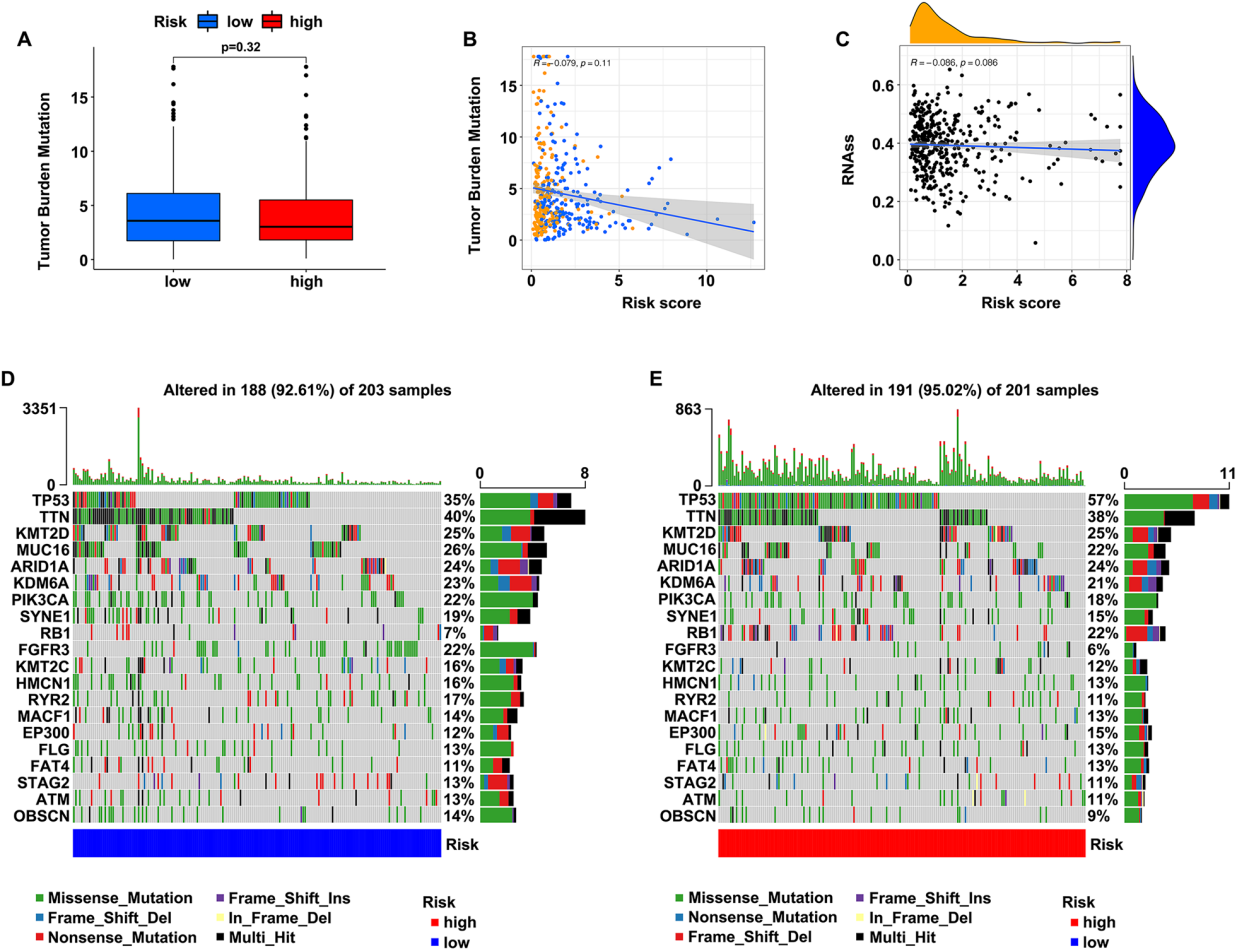
Characteristics of the SRGs-related prognostic signature. **A** Tumor mutation burden in the low- and high-risk groups. **B** Correlation between the risk score and TMB. **C** Association between the risk score and RNAss. **D**–**E** OncoPrints of the 20 high-ranking genes with the highest mutation frequency in the low- (**D**) and high-risk groups (**E**)

As reported, the tumor mutation burden (TMB) was a catalyst of tumor progression and had a close linkage with the tumor outcome, however, we did not observe any significant difference between high- and low-risk groups (Fig. 7A). Moreover, no obvious correlations were detected between the risk score and TMB (Fig. 7B), as well as the RNA stemness (RNAss) (Fig. 7C). The mafttools package was chosen for the analysis of the distribution of the somatic mutations in the risk groups, which indicated that the high-risk group experienced a little bit increased TMB (95.02%) than low-risk group (92.61%), and the most significant mutated genes were TP53 and TTN. TP53 was the gene with the highest mutation rate (low/high = 35%/57%), while TTN was the gene with mutation rate of 40% in the low-risk group and 38% in the high-risk group (Fig. 7D, E).

### Drug sensitivity evaluation of the SRGs-related prognostic signature

**Fig. 8:**
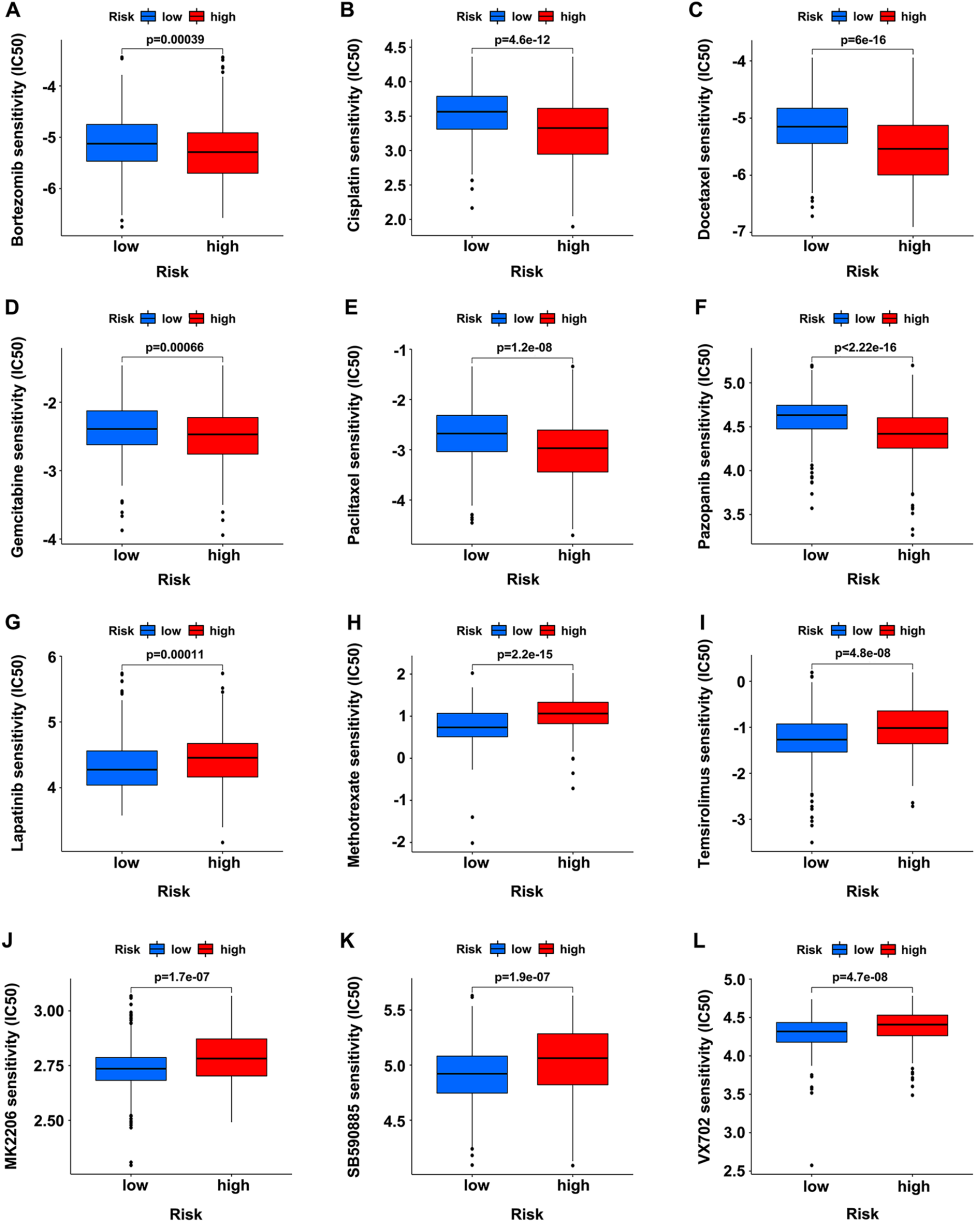
Therapeutic response evaluation with the SRGs-related prognostic signature. **A**–**F** Drug sensitivity (IC50) of bortezomib (**A**), cisplatin (**B**), docetaxel (**C**), gemcitabine (**D**), paclitaxel (**E**), pazopanib (**F**), lapatinib (**G**), methotrexate (**H**), temsirolimus (**I**), MK2206 (**J**), SB590885 (**K**), and VX702 (**L**) in the high- (red) and low-risk groups (blue)

The chemotherapeutic agents were screened based on the GDSC drug susceptibility database (https://www.cancerrxgene.org/), and the relationships between the SRGs-related prognostic signature and the responsiveness to various anticancer drugs were evaluated by calculating the half-maximal inhibitory concentration (IC50) of the samples. The high-risk group revealed more sensitive to bortezomib (Fig. 8A), cisplatin (Fig. 8B), docetaxel (Fig. 8C), gemcitabine (Fig. 8D), paclitaxel (Fig. 8E) and pazopanib (Fig. 8F), while the low-risk group was more sensitive to lapatinib (Fig. 8G), methotrexate (Fig. 8H), temsirolimus (Fig. 8I), MK2206 (Fig. 8J), SB590885 (Fig. 8K) and VX702 (Fig. 8L). In light of these findings, we believed that the risk scores based on the SRGs-related genes might be promising indicators to guide the selection of the clinical medical therapies.

## Discussion

To date, researches on various approaches aims to display the pathogenesis, identification of diagnostic, prognostic biomarkers and therapeutic targets of urinary system cancers have been conducted, which helps to facilitate and improve clinical decision-making for cancer patients. For instance, di Meo et al. suggested that lipidomics was a promising tool which should include in next decade for patient-tailored therapy perspective [26]. Detection of metabolic alterations by using multi-omics approach integrating transcriptomics, metabolomics, and lipidomics was a powerful strategy for better comprehension of cancer progression and provided potential prognostic biomarkers, as well as therapeutic applications for including prostate cancer [27, 28], renal cell carcinoma [29, 30], and bladder cancer [31, 32]. In addition, miRNAs also represented the potential to be biomarkers for the prediction of carcinogenicity or invasiveness of bladder cancer since 2010s, as well as its predictive value in discriminating NMIBC patients with cystitis or with nonmalignant hematuria, including miR-126, miR-214, miR-155, miR-20a. miR-146a-5p, miR-146, etc. [33]. As main immunotherapeutic options, programmed cell death 1 (PD1), PD1 ligand (PDL-1), and cytotoxic T-lymphocyte antigen 4 (CTLA-4) expression might be served as potential factors for individual selection of treatment with immune checkpoint inhibitors for bladder cancer patients [34, 35]. During last few decades, urinary liquid biopsy has gained growing attention about its utilization as biomarkers for prognosis and prediction of drug response, which was a non-invasive test with potential to improve the diagnostic and therapeutic pathway of bladder cancer [36]. Although with the improvement of detection approaches, prognosis prediction and medical therapy selection for bladder cancer, the patients still could not retrieve a good outcome due to the high recurrence or distant metastasis rate, and drug resistance [37–39]. Early diagnosis, personalized treatment and regular follow-up are the key to achieve and amelioration of patients’ prognosis. Thus, discovering novel biomarkers to ameliorate survival and evaluate drug response is urgently needed for bladder cancer.

Evidences have shown that SiaTs mediates tumor development through regulating events like proliferation [40], metastasis [41], angiogenesis [42] and chemotherapy resistance [43]. In the current study, we identified 17 differentially expressed SiaTs in bladder cancer, and divided patients into two SiaTs_Clusters based on their expression level. Then, two gene_Clusters were established based on the DEGs between SiaTs_Clusters, and 7 genes (CD109, TEAD4, FN1, TM4SF1, CDCA7L, ATOH8 and GZMA) were screened by LASSO and Cox regression analysis to generate the SRGs-related prognostic signature, which separated patients into high- and low-risk groups. Survival analysis suggested that patients in the low-risk group had better prognosis and longer survival time. As well, the time-dependent ROC curve was applied to evaluate the prognostic predictive value of the signature in predicting 1-, 3-, and 5-years survival rates, implying the good predictive ability of the signature that further validated with the constructed nomogram. The calibration curve for internal validation of the nomogram showed good consistency between the nomogram-predicted OS and observed OS in 1, 2, and 3 years. These findings suggested that the newly established SRGs-related prognostic signature could effectively evaluate prognosis, acting as a supplementary means for outcome assessment of bladder cancer patients.

As one essential part of the prognostic signature, TEA domain transcription factor 4 (TEAD4) is a key member of the TEAD family. It plays important roles in cancer-related processes, including epithelial to mesenchymal transition (EMT) [44], metastasis [45], vasculogenic mimicry [46], and chemoresistance [47]. Studies have demonstrated that TEAD4 mediated EMT of bladder cancer through PI3K/Akt pathway, indicating that TEAD4 could serve as an effective biomarker for prognosis prediction and a potential target for the treatment of metastatic bladder cancer [48]. In our study, TEAD4 was upregulated in the high-risk group, indicating that it might act as an oncogene in bladder cancer, which was consistent with the previous research and its positive association with the abundance of immune infiltration. It was found that TEAD4 high expression group was enriched in multiple immune related pathways, and various infiltrated immune cells were related to TEAD4 expression, revealing that it was a potential immunoregulator in bladder cancer [49]. Another critical component was ATOH8, belongs to a large superfamily of transcriptional regulators of basic helix loop helix proteins. It is not only involved in embryonic development, but also in the occurrence and development of cancer [50]. Xu et al. found that ATOH8-V1 was a novel pro-metastatic factor which enhanced cancer metastasis, and served as a potential therapeutic target for treatment of metastatic cancers [51]. Song et al. reported that ATOH8 appeared to be a tumor suppressor which induced the stem cell features and chemoresistance in hepatocellular carcinoma [52]. In the current study, ATOH8 was downregulated in the high-risk group, suggesting that it was a protective factor for bladder cancer which might due to its negative correlation with immune cell infiltration.

Studies have shown that SiaTs could promote cancer progression by affecting immune cells infiltration that played crucial roles in prognosis of cancers [53, 54]. Hence, we analyzed the abundance of immune infiltration in high- and low-risk groups which was statistically different between two groups. It was found that the CD56dim natural killer cells and monocytes were less enriched whereas 19 types of immune cells (activated B cells, activated CD4 T cells, activated CD8 T cells, activated dendritic cells, eosinophils, gamma delta T cells, immature B cells, immature dendritic cells, MDSC, macrophages, mast cells, natural killer T cells, natural killer cells, neutrophils, plasmacytoid dendritic cells, regulatory T cells, T follicular helper cells, type 1 T helper cells, and type 2 T helper cells) were more enriched in high-risk group in bladder cancer patients following with poorer outcome. Yang et al. reported that neutrophils enriched in the stroma of bladder cancer was potentially represented as a reliable maker of poor prognosis of bladder cancer patients [55]. Macrophages are believed to have close linkage with the occurrence and progression of bladder cancer [56, 57]. Jin et al. found that pan-macrophage infiltration was significantly correlated with poor prognosis of muscle-invasive bladder cancer [58]. Liu et al. found that high stromal tumor infiltrating mast cells was an independent unfavorable prognosticator for muscle-invasive bladder cancer patients [59]. The above evidences were consistent with our findings, illustrating that the impacts of SiaTs on bladder cancer might rely on the infiltration of immune cells.

As the chemotherapy and immunotherapy are the most important adjuvant treatments, we further utilized the GDSC drug sensitivity database to screen the chemotherapeutic reagents to promote personalized medication guidance for bladder cancer. According to IC50 prediction, patients in high-risk group were more sensitive to reagents like bortezomib, cisplatin, docetaxel, gemcitabine, paclitaxel and pazopanib. In addition, we compared the expressions of 25 types of immune checkpoint genes between high- and low-risk groups, finding 21 kinds were increased while only TNFRSF4 was decreased in high-risk group. It might be helpful in choosing the suitable immune checkpoint inhibitors for immuno-treatment, and guiding the selection of personalized medical therapy for bladder cancer.

## Conclusions

In summary, we identified 7 SiaTs-related genes to establish a prognostic risk model that could effectively and accurately predict the outcomes and drug sensitivity of bladder cancer patients. Also, our findings suggested the critical roles of SiaTs in bladder cancer, which might be associated with the modulation of tumor immune microenvironment. However, some limitations still existed and further researches are needed to verify the findings, as well as the molecular mechanism both in vitro and in vivo.

## Data Availability

All figures adopted in this study to support the findings are included in the article.

## Abbreviations

SiaTs: Sialyltransferases
SRGs: SiaTs-related genes
LASSO: Least absolute shrinkage and selection operator
ROC: Receiver operating characteristic
NMIBC: Non-muscle invasive bladder cancer
MIBC: Muscle-invasive bladder cancer
TCGA: The Cancer Genome Atlas
GEO: Gene Expression Omnibus
GSVA: Gene set variation analysis
ssGSEA: Single sample gene set enrichment analysis
DEGs: Differentially expressed genes
GO: Gene Ontology
KEGG: Kyoto Encyclopedia of Genes and Genomes
PCA: Principal component analysis
SRGs_score: Sialyltransferases-related gene score
AUC: Area under the curve
CNV: Copy number variations
BP: Biological process
CC: Cellular component
MF: Molecular function
OS: Overall survival
Tregs: T cells regulatory
ICIs: Immune checkpoint genes
TMB: Tumor mutation burden
RNAss: RNA stemness
PD1: Programmed cell death 1
PDL-1: PD1 ligand
CTLA-4: Cytotoxic T-lymphocyte antigen 4
TEAD4: TEA domain transcription factor 4
EMT: Epithelial to mesenchymal transition

## References

[1] Martin A, Woolbright BL, Umar S, Ingersoll MA, Taylor JA (2022). Bladder cancer, inflammageing and microbiomes. Nat Rev Urol.

[2] Rani B, Ignatz-Hoover JJ, Rana PS, Driscoll JJ (2023). Current and emerging strategies to treat urothelial carcinoma. Cancers (Basel).

[3] Yao J, Liu Y, Yang J, Li M, Li S, Zhang B (2022). Single-Cell sequencing reveals that DBI is the key gene and potential therapeutic target in quiescent bladder cancer stem cells. Front Genet.

[4] Lin W, Sun J, Sadahira T, Xu N, Wada K, Liu C (2021). Discovery and validation of nitroxoline as a novel STAT3 inhibitor in drug-resistant urothelial bladder cancer. Int J Biol Sci.

[5] Roh YG, Mun JY, Kim SK, Park W, Jeong MS, Kim TN (2020). Fanconi anemia pathway activation by FOXM1 is critical to bladder cancer recurrence and anticancer drug resistance. Cancers (Basel).

[6] Huang J, Su R, Chen Z, Jiang S, Chen M, Yuan Y (2022). The efficacy and safety of first-line treatment in cisplatin-ineligible advanced upper tract urothelial carcinoma patients: a comparison of PD-1 inhibitor and carboplatin plus gemcitabine chemotherapy. Oncoimmunology.

[7] Wang M, Chen X, Tan P, Wang Y, Pan X, Lin T (2022). Acquired semi-squamatization during chemotherapy suggests differentiation as a therapeutic strategy for bladder cancer. Cancer Cell.

[8] Zhang J, Xun M, Li C, Chen Y (2022). The O-GlcNAcylation and its promotion to hepatocellular carcinoma. Biochim Biophys Acta Rev Cancer.

[9] Yang S, Cui M, Liu Q, Liao Q (2022). Glycosylation of immunoglobin G in tumors: function, regulation and clinical implications. Cancer Lett.

[10] Li J, Guo B, Zhang W, Yue S, Huang S, Gao S (2022). Recent advances in demystifying O-glycosylation in health and disease. Proteomics.

[11] Marques C, Poças J, Gomes C, Faria-Ramos I, Reis CA, Vivès RR (2022). Glycosyltransferases EXTL2 and EXTL3 cellular balance dictates Heparan Sulfate biosynthesis and shapes gastric cancer cell motility and invasion. J Biol Chem.

[12] Liu J, Li M, Wu J, Qi Q, Li Y, Wang S (2022). Identification of ST3GAL5 as a prognostic biomarker correlating with CD8+ T cell exhaustion in clear cell renal cell carcinoma. Front Immunol.

[13] Zhu Q, Zhou H, Wu L, Lai Z, Geng D, Yang W (2022). O-GlcNAcylation promotes pancreatic tumor growth by regulating malate dehydrogenase 1. Nat Chem Biol.

[14] Wang Q, Liao C, Tan Z, Li X, Guan X, Li H (2023). FUT6 inhibits the proliferation, migration, invasion, and EGF-induced EMT of head and neck squamous cell carcinoma (HNSCC) by regulating EGFR/ERK/STAT signaling pathway. Cancer Gene Ther.

[15] Hu Q, Tian T, Leng Y, Tang Y, Chen S, Lv Y (2022). The O-glycosylating enzyme GALNT2 acts as an oncogenic driver in non-small cell lung cancer. Cell Mol Biol Lett.

[16] Lin S, Zhou L, Dong Y, Yang Q, Yang Q, Jin H (2021). Alpha-(1,6)- fucosyltransferase (FUT8) affects the survival strategy of osteosarcoma by remodeling TNF/NF-κB2 signaling. Cell Death Dis.

[17] Mabe NW, Huang M, Dalton GN, Alexe G, Schaefer DA, Geraghty AC (2022). Transition to a mesenchymal state in neuroblastoma confers resistance to anti-GD2 antibody via reduced expression of ST8SIA1. Nat Cancer.

[18] Kvorjak M, Ahmed Y, Miller ML, Sriram R, Coronnello C, Hashash JG (2020). Cross-talk between colon cells and macrophages increases ST6GALNAC1 and MUC1-sTn expression in ulcerative colitis and colitis-associated colon cancer. Cancer Immunol Res.

[19] Pietrobono S, Anichini G, Sala C, Manetti F, Almada LL, Pepe S (2020). ST3GAL1 is a target of the SOX2-GLI1 transcriptional complex and promotes melanoma metastasis through AXL. Nat Commun.

[20] Guerrero PE, Miró L, Wong BS, Massaguer A, Martínez-Bosch N, Llorens R (2020). Knockdown of α2,3-sialyltransferases impairs pancreatic cancer cell migration, invasion and e-selectin-dependent adhesion. Int J Mol Sci.

[21] Hait NC, Maiti A, Wu R, Andersen VL, Hsu CC, Wu Y (2022). Extracellular sialyltransferase st6gal1 in breast tumor cell growth and invasiveness. Cancer Gene Ther.

[22] Hao J, Zeltz C, Pintilie M, Li Q, Sakashita S, Wang T (2019). Characterization of distinct populations of carcinoma-associated fibroblasts from non-small cell lung carcinoma reveals a role for ST8SIA2 in cancer cell invasion. Neoplasia.

[23] Liu R, Cao X, Liang Y, Li X, Jin Q, Li Y (2022). Downregulation of ST6GAL1 promotes liver inflammation and predicts adverse prognosis in hepatocellular carcinoma. J Inflamm Res.

[24] Wang WY, Cao YX, Zhou X, Wei B, Zhan L, Sun SY (2019). Stimulative role of ST6GALNAC1 in proliferation, migration and invasion of ovarian cancer stem cells via the Akt signaling pathway. Cancer Cell Int.

[25] Scott E, Archer Goode E, Garnham R, Hodgson K, Orozco-Moreno M, Turner H (2023). ST6GAL1-mediated aberrant sialylation promotes prostate cancer progression. J Pathol.

[26] di Meo NA, Lasorsa F, Rutigliano M, Milella M, Ferro M, Battaglia M (2023). The dark side of lipid metabolism in prostate and renal carcinoma: novel insights into molecular diagnostic and biomarker discovery. Expert Rev Mol Diagn.

[27] Lasorsa F, di Meo NA, Rutigliano M, Ferro M, Terracciano D, Tataru OS (2023). Emerging hallmarks of metabolic reprogramming in prostate cancer. Int J Mol Sci.

[28] Lucarelli G, Loizzo D, Ferro M, Rutigliano M, Vartolomei MD, Cantiello F (2019). Metabolomic profiling for the identification of novel diagnostic markers and therapeutic targets in prostate cancer: an update. Expert Rev Mol Diagn.

[29] di Meo NA, Lasorsa F, Rutigliano M, Loizzo D, Ferro M, Stella A (2022). Renal cell carcinoma as a metabolic disease: an update on main pathways, potential biomarkers, and therapeutic targets. Int J Mol Sci.

[30] Lucarelli G, Loizzo D, Franzin R, Battaglia S, Ferro M, Cantiello F (2019). Metabolomic insights into pathophysiological mechanisms and biomarker discovery in clear cell renal cell carcinoma. Expert Rev Mol Diagn.

[31] di Meo NA, Loizzo D, Pandolfo SD, Autorino R, Ferro M, Porta C (2022). Metabolomic approaches for detection and identification of biomarkers and altered pathways in bladder cancer. Int J Mol Sci.

[32] Sahu D, Lotan Y, Wittmann B, Neri B, Hansel DE (2017). Metabolomics analysis reveals distinct profiles of nonmuscle-invasive and muscle-invasive bladder cancer. Cancer Med.

[33] Aveta A, Cilio S, Contieri R, Spena G, Napolitano L, Manfredi C (2023). Urinary MicroRNAs as biomarkers of urological cancers: a systematic review. Int J Mol Sci.

[34] Ferro M, Crocetto F, Tataru S, Barone B, Dolce P, Lucarelli G (2023). Predictors of efficacy of immune checkpoint inhibitors in patients with advanced urothelial carcinoma: a systematic review and meta-analysis. Clin Genitourin Cancer.

[35] Barone B, Calogero A, Scafuri L, Ferro M, Lucarelli G, Di Zazzo E (2022). Immune checkpoint inhibitors as a neoadjuvant/adjuvant treatment of muscle-invasive bladder cancer: a systematic review. Cancers (Basel).

[36] Ferro M, La Civita E, Liotti A, Cennamo M, Tortora F, Buonerba C (2021). Liquid biopsy biomarkers in urine: a route towards molecular diagnosis and personalized medicine of bladder cancer. J Pers Med.

[37] Pan S, Li S, Zhan Y, Chen X, Sun M, Liu X (2022). Immune status for monitoring and treatment of bladder cancer. Front Immunol.

[38] Biswas PK, Kwak Y, Kim A, Seok J, Kwak HJ, Lee M (2022). TTYH3 modulates bladder cancer proliferation and metastasis via FGFR1/H-Ras/A-Raf/MEK/ERK pathway. Int J Mol Sci.

[39] Chow PM, Dong JR, Chang YW, Kuo KL, Lin WC, Liu SH (2022). The UCHL5 inhibitor b-AP15 overcomes cisplatin resistance via suppression of cancer stemness in urothelial carcinoma. Mol Ther Oncolytics.

[40] Wang L, Chen X, Wang L, Wang S, Li W, Liu Y (2021). Knockdown of ST6Gal-I expression in human hepatocellular carcinoma cells inhibits their exosome-mediated proliferation- and migration-promoting effects. IUBMB Life.

[41] Nguyen K, Yan Y, Yuan B, Dasgupta A, Sun J, Mu H (2018). ST8SIA1 regulates tumor growth and metastasis in TNBC by activating the FAK-AKT-mTOR signaling pathway. Mol Cancer Ther.

[42] Yeo HL, Fan TC, Lin RJ, Yu JC, Liao GS, Chen ES (2019). Sialylation of vasorin by ST3Gal1 facilitates TGF-β1-mediated tumor angiogenesis and progression. Int J Cancer.

[43] Ma H, Zhou H, Song X, Shi S, Zhang J, Jia L (2015). Modification of sialylation is associated with multidrug resistance in human acute myeloid leukemia. Oncogene.

[44] Zhang W, Li J, Wu Y, Ge H, Song Y, Wang D (2018). TEAD4 overexpression promotes epithelial-mesenchymal transition and associates with aggressiveness and adverse prognosis in head neck squamous cell carcinoma. Cancer Cell Int.

[45] Cozzaglio M, Ceschi S, Groaz E, Sturlese M, Sissi C (2022). G-quadruplexes formation within the promoter of TEAD4 oncogene and their interaction with Vimentin. Front Chem.

[46] Zhang Y, Bai J, Cheng R, Zhang D, Qiu Z, Liu T (2022). TAZ promotes vasculogenic mimicry in gastric cancer through the upregulation of TEAD4. J Gastroenterol Hepatol.

[47] He L, Yuan L, Sun Y, Wang P, Zhang H, Feng X (2019). Glucocorticoid receptor signaling activates TEAD4 to promote breast cancer progression. Cancer Res.

[48] Chi M, Liu J, Mei C, Shi Y, Liu N, Jiang X (2022). TEAD4 functions as a prognostic biomarker and triggers EMT via PI3K/AKT pathway in bladder cancer. J Exp Clin Cancer Res.

[49] Wang J, Shen C, Zhang J, Zhang Y, Liang Z, Niu H (2021). TEAD4 is an immune regulating-related prognostic biomarker for bladder cancer and possesses generalization value in pan-cancer. DNA Cell Biol.

[50] Divvela SSK, Saberi D, Brand-Saberi B (2022). Atoh8 in development and disease. Biology (Basel).

[51] Xu M, Huang S, Dong X, Chen Y, Li M, Shi W (2021). A novel isoform of ATOH8 promotes the metastasis of breast cancer by regulating RhoC. J Mol Cell Biol.

[52] Song Y, Pan G, Chen L, Ma S, Zeng T, Man Chan TH (2015). Loss of ATOH8 increases stem cell features of hepatocellular carcinoma cells. Gastroenterology.

[53] Hu X, Yang Y, Wang Y, Ren S, Li X (2022). Identifying an immune-related gene ST8SIA1 as a novel target in patients with clear-cell renal cell carcinoma. Front Pharmacol.

[54] Ko CY, Chu TH, Hsu CC, Chen HP, Huang SC, Chang CL (2022). Bioinformatics analyses identify the therapeutic potential of ST8SIA6 for colon cancer. J Pers Med.

[55] Yang M, Wang B, Hou W, Yu H, Zhou B, Zhong W (2022). Negative effects of stromal neutrophils on T cells reduce survival in resectable urothelial carcinoma of the bladder. Front Immunol.

[56] Tang Z, Tang C, Sun C, Ying X, Shen R (2022). M1 macrophage-derived exosomes synergistically enhance the anti- bladder cancer effect of gemcitabine. Aging (Albany NY).

[57] Chiang Y, Tsai YC, Wang CC, Hsueh FJ, Huang CY, Chung SD (2022). Tumor-Derived C-C motif ligand 2 induces the recruitment and polarization of tumor-associated macrophages and increases the metastatic potential of bladder cancer cells in the postirradiated microenvironment. Int J Radiat Oncol Biol Phys.

[58] Jin S, Zeng H, Liu Z, Jin K, Liu C, Yan S (2022). Stromal tumor-associated macrophage infiltration predicts poor clinical outcomes in muscle-invasive bladder cancer patients. Ann Surg Oncol.

[59] Liu Z, Zhu Y, Xu L, Zhang J, Xie H, Fu H (2018). Tumor stroma-infiltrating mast cells predict prognosis and adjuvant chemotherapeutic benefits in patients with muscle invasive bladder cancer. Oncoimmunology.

